# Economic Impact of Respiratory Syncytial Virus Infections in Children Under 5 Years of Age Attending Primary Care in Italy: A Prospective Cohort Study in Two Regions

**DOI:** 10.1111/irv.70074

**Published:** 2025-02-02

**Authors:** Valérie D. V. Sankatsing, Jojanneke van Summeren, Fasika Molla Abreha, Elisabetta Pandolfi, Maria Chironna, Daniela Loconsole, Rolf Kramer, John Paget, Caterina Rizzo

**Affiliations:** ^1^ Department of Infectious Diseases in Primary Care Nivel, Netherlands Institute for Health Services Research Utrecht Netherlands; ^2^ Predictive and Preventive Medicine Research Unit Bambino Gesù Children's Hospital, IRCCS Rome Italy; ^3^ Department of Interdisciplinary Medicine University of Bari Bari Italy; ^4^ Sanofi Vaccines Sanofi Lyon France; ^5^ Department of Translational Research and New Technologies in Medicine and Surgery University of Pisa Pisa Italy

**Keywords:** costs, outpatient, primary care, RSV, work absence

## Abstract

**Background:**

Accurate cost estimates of respiratory syncytial virus (RSV) infections in primary care are limited, despite the majority of cases being managed in this setting. This study aims to estimate healthcare costs for children with RSV in primary care and the related costs of parental work absence.

**Methods:**

Children < 5 years of age with symptoms of acute respiratory infections were recruited via primary care paediatricians in two Italian regions for a prospective cohort study on the RSV burden in primary care, during the 2019/2020 winter. Healthcare utilization, medication use and parental work absence were assessed during a 14‐day follow‐up period. Average costs were estimated per RSV episode for the overall study population, as well as per age group.

**Results:**

Two hundred ninety three children were recruited, of which 119 tested RSV positive (41%) and 109 were included. In total, 89% of RSV‐positive children (97/109) had ≥ 1 repeat paediatrician visit(s), and 10% (11/109) visited the ED. The mean number of repeat visits was 3.8 (SD: 4.0) and the mean duration of work absence 4.0 days (SD: 5.0). Average costs per RSV episode were €730 (95% CI: €691–€771), with direct medical costs accounting for 25% (€183 [95% CI: €174–€191]) and indirect costs related to work absence for 75% (€547 [95% CI: €509–€587]).

**Conclusions:**

Costs associated with RSV infections in young children in primary care are considerable due to a substantial number of paediatrician visits and high rates of parental work absence. These costs are important to include in decision‐making regarding the implementation of new RSV immunization strategies in national immunization programmes.

**Abbreviations**
95% CI: 95% confidence interval
ARI: acute respiratory infection
ED: emergency department
RSV: respiratory syncytial virus
RT‐PCR: real‐time polymerase chain reaction
SD: standard deviation

## 1 Introduction

Respiratory syncytial virus (RSV) is the most common pathogen causing respiratory diseases in young children [1, 2]. Before reaching the age of 1 year, approximately 60%–70% of all children have experienced an RSV infection, and nearly all children are infected before the age of two [2, 3, 4]. In Western countries, mortality due to an RSV infection is rare; however, RSV infections in infants and young children have a substantial impact on the healthcare system [2, 5, 6]. Furthermore, RSV infections in young children can significantly disrupt parental activities, leading to work absences and reduced productivity [7, 8, 9].

To prevent RSV infections and reduce the associated burden, the European Medicines Agency has recently approved a maternal RSV vaccine and a long‐acting monoclonal antibody (mAb), both of which provide passive immunization against RSV [10, 11, 12, 13, 14]. Data on the economic impact of RSV infections are essential to guide the optimal use of these new prophylactic immunization strategies and to evaluate their cost‐effectiveness.

Several studies have assessed the costs associated with RSV‐related hospitalizations in young children [15, 16]. There is, however, a lack of research specifically focused on costs within primary care settings, while the majority of children with RSV are managed in primary care [17, 18]. In addition, there is limited information on the indirect costs, for example, work absence incurred by parents caring for children with an RSV infection. A systematic literature review on the overall costs per RSV episode in children under 5 years old estimated an average global cost of €3452 per RSV episode for inpatient management and €299 for outpatient management [6]. However, only 7% of cases in this review were outpatients, that is, recruited in primary care or emergency departments (EDs). A more recent study by Diez‐Gandia et al., which primarily included children recruited through primary care, estimated that the median direct healthcare costs per RSV episode were about €600 in Spain, though indirect costs were not reported [19].

Our study aimed to assess the direct medical costs of RSV‐infected young children managed in primary care in two regions in Italy, as well as the indirect costs associated with parental work absence due to the child's RSV illness. Primary care organization varies across Europe. In Italy, paediatric primary care is typically free of charge for all children, encompassing visits, basic diagnostics and prescriptions [20]. For children under 6 years of age, primary care is exclusively delivered by primary care paediatricians [21]. This study was based on data collected by paediatricians in the RSV ComNet study, just before the COVID‐19 pandemic emerged [22, 23].

## 2 Methods

### 2.1 Study Protocol

In the RSV ComNet study, children under the age of 5 years who consulted a primary care paediatrician with symptoms of an acute respiratory infection (ARI)—as defined by the WHO case definition—were eligible for RSV testing [24]. The RSV ComNet study protocol is published in more detail elsewhere [22]. Children were recruited in the winter season 2019/2020 by 24 paediatricians in two Italian municipalities: Rome (Lazio region) with 169,969 and Bari (Puglia region) with 48,652 inhabitants under 5 years of age in 2019 [25]. The aim was to enroll approximately 400 children under 5 years of age with ARI symptoms in total [23].

At the day of consultation (Day 1), paediatricians collected a nasopharyngeal swab and completed a short clinical report about patient demographics, medical history and clinical symptoms (Figure S1). The swabs were tested in medical diagnostic laboratories using a commercial multiplex real‐time polymerase chain reaction (RT‐PCR; Allplex Respiratory Full Panel Assay) for 16 viruses (including adenovirus, influenza A and B, Parainfluenza 1–4, RSV A and B, metapneumovirus, coronavirus OC43, 229E and NL63, rhinovirus, bocavirus and enterovirus). A follow‐up phone questionnaire was conducted with the parents of children with a laboratory‐confirmed RSV diagnosis, approximately 14 days after the swab collection (Day‐14 questionnaire). Exclusion criteria included parents who had insufficient knowledge of the national language or intellectual disabilities that hindered their ability to complete the questionnaire.

### 2.2 Outcome Measurements

Healthcare resources utilized in relation to the RSV infection, including the number of paediatrician visits, emergency department (ED) visits, hospital admissions and medication used (bronchodilators, corticosteroids, antibiotics, over‐the‐counter pain medication, nasal spray and cough syrup), were measured with the Day‐14 questionnaire. Paediatrician visits in this study were categorized into initial and repeat paediatrician visits. Repeat visits encompassed both regular consultations to paediatricians, phone/email contacts, home visits and out‐of‐hours services by paediatricians.

In addition to healthcare resource utilization, the questionnaire assessed parental work absence and children's absences from school or day care during the 14‐day period between swab collection and questionnaire completion. Because we have no follow‐up data beyond the time horizon of the questionnaire, the maximum duration of work absence and school/day‐care absence was limited to 14 days in this study.

### 2.3 Analysis

Average costs per RSV episode (i.e., costs per child with an RSV infection) were calculated from two perspectives:

1. Healthcare sector perspective: this included direct medical costs related to the management of RSV in the primary care setting, covering expenses associated with paediatrician visits (including out‐of‐hours services), emED visits and medications (including nonreimbursed medications). Children who were hospitalized were excluded from the analysis (Figure S1), since the focus of this study is costs incurred in primary care settings, and other studies with larger sample sizes of hospitalized children have already provided insights into the associated costs in hospital settings [15, 16].
2. Societal perspective: this encompassed both the direct medical costs and the indirect costs associated with parental work absence.

Outcomes were calculated for the overall study population as well as stratified by age (0–11, 12–23 and 24–59 months). Demographics were summarized by frequency (%) for categorical variables and by mean and standard deviation (SD) for continuous variables. Descriptive statistics were conducted using Stata 16.

Costs associated with parental work absence were calculated based on the mean number of days of absence reported in the Day‐14 questionnaire and the average daily wage in Italy. An overview of all unit costs, other input and their sources is listed in Table S1. Confidence intervals (95% CI) around cost estimates were calculated with bootstrapping using R 4.3.1. The R boot package was used to perform bootstrap resampling (10,000 samples) [26]. Costs were converted to 2020 price levels in Euros using the consumer price index.

## 3 Results

Between Weeks 45/2019 and 12/2020, 293 children were tested for RSV (Figure S1). In total, 119 children tested positive for RSV (41%), with a median age of 15 months (Table 1). The majority of children (95%) were term‐born (≥ 37 weeks of gestation) and had no major comorbidity (98%). Other baseline demographics, clinical characteristics and virological test results are shown in Table 1.

**TABLE 1:** Baseline demographics, clinical characteristics and test results for all RSV‐positive children^a^ and by age.

	Total (*n* = 119)	0–11 months (*n* = 53)	12–23 months (*n* = 26)	24–59 months (*n* = 40)
Demographics				
Boys (*n*, %)	59 (50%)	30 (57%)	13 (50%)	16 (40%)
Age in months median (IQR)	15 (7–30)	6 (4–9)	20 (15–21)	35 (30–46)
Clinical symptoms				
Cough (*n*, %)	117 (98%)	52 (98%)	26 (100%)	39 (98%)
Coryza (*n*, %)	106 (89%)	46 (87%)	26 (100%)	34 (85%)
Shortness of breath (*n*, %)	89 (76%)	45 (87%)	15 (60%)	29 (73%)
Sore throat (*n*, %)	36 (30%)	16 (30%)	10 (38%)	10 (25%)
Days of symptoms before sample uptake (median, IQR)	3 (2–4)	3 (2–5)	3 (2–4)	2 (1.5–4)
Relevant medical history				
Prematurity	6 (5%)	0	4 (15%)	2 (5%)
Other chronic medical condition	2 (2%)	0	0	2 (5%)
Chronic respiratory disease	1 (1%)	0	0	1 (3%)
Malnutrition	0	0	0	0
Virological test results				
RSV A	91 (76%)	40 (75%)	21 (81%)	30 (75%)
RSV B	28 (24%)	13 (25%)	5 (19%)	10 (25%)
Co‐infection with at least one other respiratory virus^b^	61 (51%)	26 (49%)	14 (54%)	21 (53%)

^a^ Including those that were lost to follow‐up (*n* = 3).

^b^ Samples were tested for 16 coviruses (see Section 2): 39 children were coinfected with 1 virus, and 22 were coinfected with two or more viruses. Rhinovirus is the most frequently measured co‐infection (*n* = 36) followed by enterovirus (*n* = 7) and adenovirus (*n* = 5).

The Day‐14 questionnaire was completed by parents of 116 RSV‐positive children (97%). In total, seven RSV‐positive children were excluded due to hospitalization (6%), leading to 109 RSV episodes used for the analysis. In our study, each RSV‐positive child represents a single RSV episode, meaning none of the included children experienced more than one RSV episode during the study period. The mean duration of illness among those included was 8.2 days (SD: 3.7) and was similar across age groups (Table 2).

**TABLE 2:** Healthcare resource utilization and societal impact in the 14 days following sample uptake.

	All ages (*n* = 109)	0–11 months (*n* = 46)	12–23 months (*n* = 25)	24–59 months (*n* = 38)
Duration of illness (days) mean ± SD	8.2 ± 3.7	8.3 ± 3.7	8.0 ± 4.2	8.0 ± 3.6
Healthcare utilization				
≥ 1 repeat paediatrician visit^a^ *n* (%)	97 (89)	45 (98)	22 (88)	30 (79)
Repeat paediatrician visit^a^ mean ± SD	3.8 ± 4.0	4.3 ± 4.1	3.8 ± 4.5	3.1 ± 3.3
Out‐of‐hours service visits *n* (%)	15 (14%)	5 (11%)	0 (0%)	10 (26%)
Emergency department visits n (%)	11 (10%)	5 (11%)	3 (12%)	3 (8%)
Medication use				
Prescribed				
Bronchodilators *n* (%)	41 (38%)	16 (35%)	13 (52%)	12 (32%)
Antibiotics *n* (%)	32 (29%)	7 (15%)	10 (40%)	15 (39%)
Corticosteroid inhalers *n* (%)	14 (13%)	5 (11%)	5 (20%)	4 (11%)
Systemic corticosteroids *n* (%)	4 (4%)	2 (4%)	1 (4%)	1 (3%)
Over‐the‐counter				
Paracetamol *n* (%)	45 (41%)	13 (28%)	16 (64%)	16 (42%)
NSAIDs *n* (%)	4 (4%)	0 (0%)	0 (0%)	4 (11%)
Other medication^b^ *n* (%)	17 (16%)	9 (20%)	4 (16%)	4 (11%)
Day‐care/preschool absence				
Absent for ≥ 1 day *n* (%)^c^	52 (48%)	4 (8.7%)	17 (68%)	31 (82%)
Duration of absence mean ± SD^d^	4.4 ± 5.4	0.9 ± 3.2	6.0 ± 5.3	7.6 ± 5.1
Parental work absence				
Absent for ≥ 1 day (*n*, %)	57 (52%)	24 (52%)	13 (52%)	20 (53%)
Duration of absence (days) mean ± SD^e^	4.0 ± 5.0	4.3 ± 5.2	4.1 ± 5.4	3.7 ± 4.5

*Note:* The maximum duration of illness, school/day‐care absence and work absence was set at 14 days (time horizon of the Day‐14 questionnaire).

Abbreviations: NSAID: nonsteroid anti‐inflammatory drug; RSV: respiratory syncytial virus; SD: standard deviation.

^a^ Encompassing regular visits (45%), consultations by phone/email (54%) and home visits (1%).

^b^ Including nasal spray and cough syrup.

^c^ Twenty‐three percent of children did not attend day care or preschool regularly; 72% of these were aged between 0 and 11 months.

^d^ Among all children (*n* = 109), including those that did not attend any facilities.

^e^ Among all parents of children included in the study, including those that did not report being absent from work.

### 3.1 Healthcare Sector Costs

**FIGURE 1:**
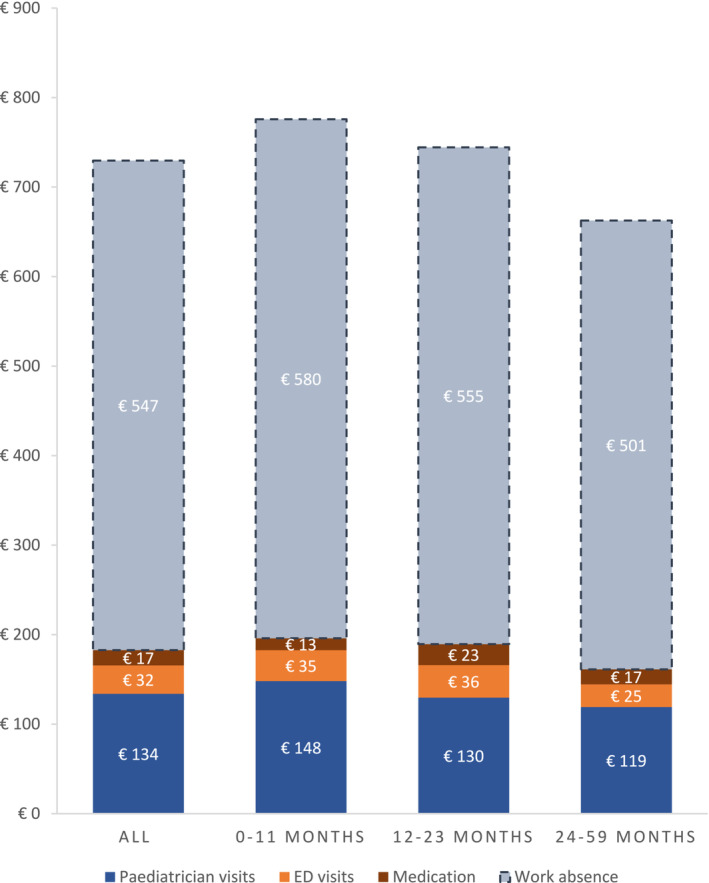
Detailed breakdown of costs (€) per RSV episode in primary care. The figure displays direct costs associated with healthcare utilization (paediatrician visits; ED visits; medication) and indirect costs related to work absence (dashed lines); primary care paediatrician visits include initial visits, repeat visits and out‐of‐hours visits; N.B. the maximum duration of work absence was set at 14 days (time horizon of the parental questionnaire). Abbreviations: ED, emergency department.

From the healthcare sector perspective, average costs per RSV episode at the primary care level were €183 (95% CI: €174–€191) (Table 3). The vast majority of these costs (73%) were attributed to visits to the primary care paediatrician, a pattern that remained consistent across different age groups (Figure 1, Table S2). The mean number of repeat paediatrican visits per RSV episode was 3.8 (SD: 4.0, Table 2). In total, 89% (97/109) of RSV‐positive children had at least one repeat visit to the paediatrician, and 10% (11/109) visited the ED (Table 2). Compared to other age groups, infants under the age of 1 year demonstrated the highest number of repeat paediatrician visits, consequently leading to slightly higher healthcare costs per RSV episode in this particular group (€196 [95% CI: €187–€205]) (Tables 2, 3). Across all age groups, bronchodilators were mostly prescribed (38%), followed by antibiotics (29%), corticosteroid inhalers (13%) and systemic corticosteroids (4%) (Table 2). Over‐the‐counter medication primarily comprised paracetamol (41%). When compared to healthcare visits, costs associated with medication contributed marginally to overall healthcare costs in all age categories (7%–12% of total healthcare sector costs, Figure 1, Table S2).

**TABLE 3:** Average costs (€) per RSV episode.

	Healthcare sector perspective (95% confidence interval)	Societal perspective (95% confidence interval)
All ages (*n* = 109)	€183 (174–191)	€730 (691–771)
0–11 months (*n* = 46)	€196 (187–205)	€776 (735–819)
12–23 months (*n* = 25)	€189 (180–199)	€744 (703–789)
24–59 months (*n* = 38)	€161 (154–169)	€663 (628–700)

Abbreviation: 95% CI: 95% confidence interval.

### 3.2 Societal Costs

Parents of 25 out of 109 children (23%) reported that their child did not have regular attendance at school or day‐care centres, the majority of which (18, 72%) belonged to the 0–11‐month age group (Table S3). The mean duration of school or day‐care absence (4.4 [SD: 5.4]) varied considerably across age groups, with infants having relatively short absences and older children experiencing considerable absence (Table 2). School and day‐care absence were not included in cost calculations.

In total, 52% of parents (57/109) was absent from work for at least 1 day, and this proportion was similar across age groups (Table 2). The mean duration of parental work absence was 4.0 days (SD: 5.0) and was consistent between age groups, despite the variations in school and day‐care absence among the different age groups.

From the societal perspective, average costs per RSV episode in primary care were €730 (95% CI: €691–€771) (Table 3). Societal costs varied slightly across different age groups, with the highest expenditures observed for the 0–11 months old (Table 3). Indirect costs associated with work absence accounted for the substantial portion of societal costs (75%, €547 [95% CI: €509–€587]), outweighing direct healthcare costs (Figure 1, Tables 3, S4). This finding was consistent across all age demographics.

## 4 Discussion

This study shows that costs associated with RSV infections in children managed in primary care are substantial, not just in the first year of life but also persisting up to the age of five. At the healthcare level, these costs are primarily driven by the substantial number of paediatrician visits. Additionally, our findings indicate that the majority of societal costs arise from parental work absence due to RSV infection.

Although unit costs of healthcare resources in primary care are notably lower than those in hospitals, our findings indicate a relatively high utilization of primary care resources during an RSV episode, resulting in significant costs. Similar patterns have also been observed in studies focusing on influenza‐like illness [27, 28]. Accurate estimates of the incidence of RSV in primary care are currently limited [29], complicating population‐based cost calculations. A recent study in Italy assessing the burden of bronchiolitis in children aged ≤ 24 months reported an incidence of 47 per 1000 person years [30]. These findings, along with ours, highlight the substantial contribution of primary care–related costs to the total economic impact of RSV.

In our study, the majority of RSV‐positive children were infected with the RSV A subtype (76%), whereas a relatively small proportion (24%) was infected with RSV B. Due to the limited number of RSV B cases, we did not stratify costs per RSV episode by subtype. However, a separate analysis on the clinical burden of RSV using the same dataset previously reported that RSV B was associated with increased healthcare utilization compared to RSV A (OR: 5.4, 95% CI: 1.2–24.6) [23]. These finding suggests that costs for RSV B episodes in our study population might be higher than for RSV A episodes but as the results were based on a small sample size for RSV B subtype, they should be interpreted with caution. In contrast, a recent literature review found no evidence to support differences in clinical severity between RSV A and RSV B [31], underscoring the need for further research to clarify potential differences in healthcare resource utilization and associated costs between the two RSV subtypes.

Average medical costs of RSV infections in children under 5 years of age managed in primary care estimated in this study are €183 (95% CI: €174–€191) per RSV episode. A small multi‐country cohort study (*n* = 83) including children from four European countries (Spain, Finland, the Netherlands and the United Kingdom) reported a considerably lower estimate of €58 per RSV episode for children who received primary care only [7]. This discrepancy likely arises from variations in healthcare resource utilization among different countries. Additionally, differences in outcomes may be attributed to distinct study designs.

Our study shows a rather high mean number of paediatrician consultations per RSV episode (1 initial visit plus a mean of 3.8 repeat visits). Similarly, a Spanish prospective multicentre study reported an average of 4.9 paediatrician visits per RSV patient [19]. It is important to note that this Spanish study also included data from hospitalized children with RSV infections. Furthermore, the participants were all under 2 years old.

Although comparisons across studies are challenging, contrasting our estimates with estimates for other prevalent childhood infections in Europe is important to grasp the full extent of the RSV burden. The largest portion of societal costs in this study was attributed to costs related to parental work absence, a pattern similarly observed for childhood influenza [27]. A prospective cohort study in the community setting conducted in the Netherlands among infants with severe acute gastroenteritis due to rotavirus or norovirus infections reported lower rates of healthcare utilization and parental work loss for children aged < 5 years compared to what we observe for RSV [32].

The data for this study were collected during the 2019/2020 RSV season, prior to the emergence of the COVID‐19 pandemic. We believe these estimates remain valuable in the postpandemic era, as recent post‐pandemic data from Italy show a similar RSV positivity rate to that reported in our study [33].

Given the rollout of first monoclonal antibody prophylaxis programmes in Europe, it is important to explore the costs of RSV in primary care for multiple countries and RSV seasons, to validate our outcomes that are currently based on data from a single country. To assess the economic disease burden of RSV infections in primary care settings across multiple European countries and RSV seasons, the data collection of RSV ComNet was continued from 2020 to 2024 in six European countries, namely, Belgium, France, Italy, the Netherlands, Spain and the United Kingdom.

Our findings hold significant value in light of recent advancements regarding the implementation of prophylactic immunization strategies. Our primary care cost estimates are crucial for refining mathematical models used in economic evaluations of mAbs and maternal vaccines, optimizing their deployment and assessing cost‐effectiveness. Decision‐makers will rely on these evaluations to guide integration of these novel interventions into national immunization programmes. An important strength of this study is that we had individual patient‐level data on both healthcare resource use and work absence among parents, considerably limiting the number of assumptions that needed to be made. Moreover, all children with ARI symptoms visiting the participating paediatricians were tested for RSV, and there was very little loss to follow‐up, largely eliminating selection bias.

Our study also has limitations. First, only short‐term costs, incurred within 14 days after swabbing, are considered in the analysis. However, a prospective multicenter RSV surveillance study showed that 92% of children with an RSV infection return to normal daily activities within this time period [19], implying that we likely capture the majority of primary care–related costs. Second, in cases were children had an RSV coinfection (51%), we could not definitively attribute the ARI symptoms and associated disease burden to RSV. However, a recently published meta‐analysis found no relation between viral coinfections and severity of RSV disease, except in cases of coinfections with hMPV [34]. Further, societal costs in our study only stem from parental work absence as information on other factors contributing to indirect costs (e.g., transportation to primary care facilities) was not available from the questionnaire. Finally, the sample size of this study is relatively small as it is based on data collected from 24 paediatricians across only two Italian regions: Lazio (Central Italy) and Puglia (Southern Italy). A separate analysis of the same dataset from the Italian arm of the RSV ComNet study (2019/2020 RSV season) previously reported variations in healthcare utilization between these two regions, specifically in the number of paediatrician visits and the proportion of ED visits [23]. While this may limit the generalizability of our cost estimates, it also highlights the inherent challenge posed by regional differences in (primary) healthcare systems. For example, a recent analysis of the Italian RSV ComNet data from the 2022/2023 season, which included broader regional coverage across five regions in Italy, found significant regional variation in antibiotic use in Italy [35]. These findings underscore the importance of considering differences in primary care system organization and associated RSV‐related costs when assessing the cost‐effectiveness of RSV immunization programmes, both within and between countries.

## 5 Conclusions

This study shows that RSV infections in children < 5 years managed within primary care settings in Italy lead to substantial costs, which are nearly as significant beyond the first year of life as during infancy. RSV infections in primary care pose a considerable economic burden at both the healthcare and the societal level, emphasizing that these costs should not be overlooked when considering infant RSV immunization. Although our findings may not directly reflect RSV‐related primary care costs in other (European) countries, they underscore the significant socio‐economic burden of RSV cases managed in primary care settings.

## Author Contributions

**Valérie D.V. Sankatsing:** conceptualization, writing – original draft, formal analysis, data curation, writing – review and editing, visualization, methodology. **Jojanneke van Summeren:** conceptualization, funding acquisition, writing – original draft, writing – review and editing, visualization, formal analysis, data curation, supervision, methodology, project administration. **Fasika Molla Abreha:** conceptualization, writing – review and editing, methodology. **Elisabetta Pandolfi:** investigation, writing – review and editing. **Maria Chironna:** investigation, writing – review and editing. **Daniela Loconsole:** investigation, writing – review and editing. **Rolf Kramer:** conceptualization, methodology, writing – review and editing. **†John Paget:** conceptualization, methodology, funding acquisition, writing – review and editing, supervision, project administration. **Caterina Rizzo:** investigation, conceptualization, methodology, writing – review and editing.

## Ethics Statement

The medical ethical committee of Bambino Gesù Children's Hospital (OPBG) in Italy provided a waiver for ethical approval (Prot. N 1301).

## Consent

Informed consent of parents of all included children was collected by the paediatrician.

## Conflicts of Interest

JvS, VS and JP declare that Nivel has received unrestricted research grants from WHO, Sanofi, AstraZeneca and the Foundation for Influenza Epidemiology. JP received a grant from the Respiratory Syncytial Virus Consortium in Europe (RESCEU) project of the ‘Innovative Medicines Initiative 2 Joint Undertaking’ grant agreement No 116019. This Joint Undertaking gets support from the ‘European Union's Horizon 2020 research and innovation programme’ and the ‘European Federation of Pharmaceutical Industries and Associations’. Jvs and JP received a grant from the Preparing for RSV Immunisation and Surveillance in Europe (PROMISE) project of the ‘Innovative Medicines Initiative 2 Joint Undertaking’ grant agreement No 101034339. This Joint Undertaking gets support from the ‘European Union's Horizon 2020 research and innovation programme’ and the ‘European Federation of Pharmaceutical Industries and Associations’. CR declares that she received fees for participation in advisory boards from Astra‐Zeneca, Seqirus, MSD, Sanofi and GSK and for CME lectures from Seqirus, Sanofi, Astra‐Zeneca, MSD and GSK. RK is an employee of Sanofi and may hold shares and/or stock options in the company. MC, DL, FMA and EP have nothing to declare.

### Peer Review

The peer review history for this article is available at https://www.webofscience.com/api/gateway/wos/peer‐review/10.1111/irv.70074.

## Supporting information


**Table S1.** Unit costs.
**Table S2.** Proportion of different cost items of total healthcare sector costs by age.
**Table S3.** School/day‐care absence.
**Table S4.** Breakdown of societal costs by age.
**Figure S1.** Patient flowchart.

## Data Availability

After termination of the ComNet RSV project, anonymized data are available on reasonable request. Inquiries can be sent to the corresponding author.
