# Supplementary material for: Economic Impact of Respiratory Syncytial Virus Infections in Children Under 5 Years of Age Attending Primary Care in Italy: A Prospective Cohort Study in Two Regions
Source: Influenza Other Respir Viruses. 2025 Feb 2;19(2):e70074. doi: 10.1111/irv.70074 (PMC11788539; doi:10.1111/irv.70074)
Supplement: Supplementary file 1 — Table S1. Unit costs. Table S2. Proportion of different cost items of total healthcare sector costs by age. Table S3. School/day‐care absence. Table S4. Breakdown of societal costs by age. Figure S1. Patient flowchart. [file IRV-19-e70074-s001.docx]

# Supporting Information

**Table 1**. Unit costs

| **Healthcare resource** | **Unit cost (€)** | **Reference** |
| --- | --- | --- |
| Healthcare utilization | | |
| Initial visit to pediatrician | €26.06 ^a^ | [1] |
| Repeat visit (regular) |  |  |
| Repeat consultation phone/email |  |  |
| Subsequent home visit |  |  |
| Out-of-hours service |  |  |
| Emergency department visit | €287.74 | [2] |
| Medication (cost per pack/unit) | | |
| *Prescribed medication ^b^* | | |
| Bronchodilators | €3.91 | [3] |
| Antibiotics | €3.27 |  |
| Corticosteroid inhalers | €27.88 |  |
| Systemic corticosteroids | €1.33 |  |
| Dispensing fee (per item) | €7.74 | [4] |
| Over-the-counter medication | | |
| *Paracetamol* | €6.45 | [5] |
| NSAID | €13.20 |  |
| Nasal spray | €13.50 |  |
| Cough syrup | €15.30 |  |
| Work absence | | |
| Daily salary in Italy ^c^ | €132.80 | [6] |

^a^ For all types of repeat visits (regular visits, home visits etc.) the same unit cost was used, as primary care paediatricians in Italy are paid based on the number of children they follow rather than per consultation type (C. Rizzo, personal communication, 2023).
^b^ Based on the most commonly prescribed bronchodilator, antibiotic and corticosteroids in this study.
^c^ Calculated based on mean annual gross earnings (before any tax deductions and social security contributions) for males and females combined assuming 262 paid workdays per year.
N.B. Costs were inflated to Euro (€) year 2020.

**Table 2.** Proportion of different cost items of total healthcare sector costs by age

|  | Paediatrician visits* | ED visits | Medication |
| --- | --- | --- | --- |
| All | 73% | 17% | 9% |
| 0-11 months | 76% | 18% | 7% |
| 12-23 months | 68% | 19% | 12% |
| 24-59 months | 74% | 16% | 10% |

*Including initial visit, repeat visits (regular), repeat consultations through phone or email, home visits and out-of-hours services
Abbreviations: ED, emergency department.

**Table 3.** School/day-care absence

*All ages*

| **Number of days of day-care/school absence** | **Frequency** | **Percentage** |
| --- | --- | --- |
| 0 | 32 | 29.4 |
| 2 | 1 | 0.9 |
| 3 | 1 | 0.9 |
| 3.5 | 1 | 0.9 |
| 4 | 2 | 1.8 |
| 5 | 6 | 5.5 |
| 6 | 1 | 0.9 |
| 7 | 14 | 12.8 |
| 10 | 8 | 7.3 |
| 14 | 18 | 16.5 |
| Infrequent day-care attendance | 25 | 22.9 |

*Children aged 0-11 months*

| **Number of days of day-care/school absence** | **Frequency** | **Percentage** |
| --- | --- | --- |
| 0 | 24 | 52.2 |
| 4 | 1 | 2.2 |
| 10 | 1 | 2.2 |
| 14 | 2 | 4.3 |
| Infrequent day-care attendance | 18 | 39.1 |

**Table 4.** Breakdown of societal costs by age

|  | **All healthcare costs (direct costs)** | **Work absence (indirect costs)** |
| --- | --- | --- |
| All ages | 25% | 75% |
| 0-11 months | 25% | 75% |
| 12-23 months | 25% | 75% |
| 24-59 months | 24% | 76% |

*Including initial visit, repeat visits (regular), repeat consultations through phone or email, home visits and out-of-hours services
Abbreviations: ED, emergency department.

**Figure 1.** Patient flowchart

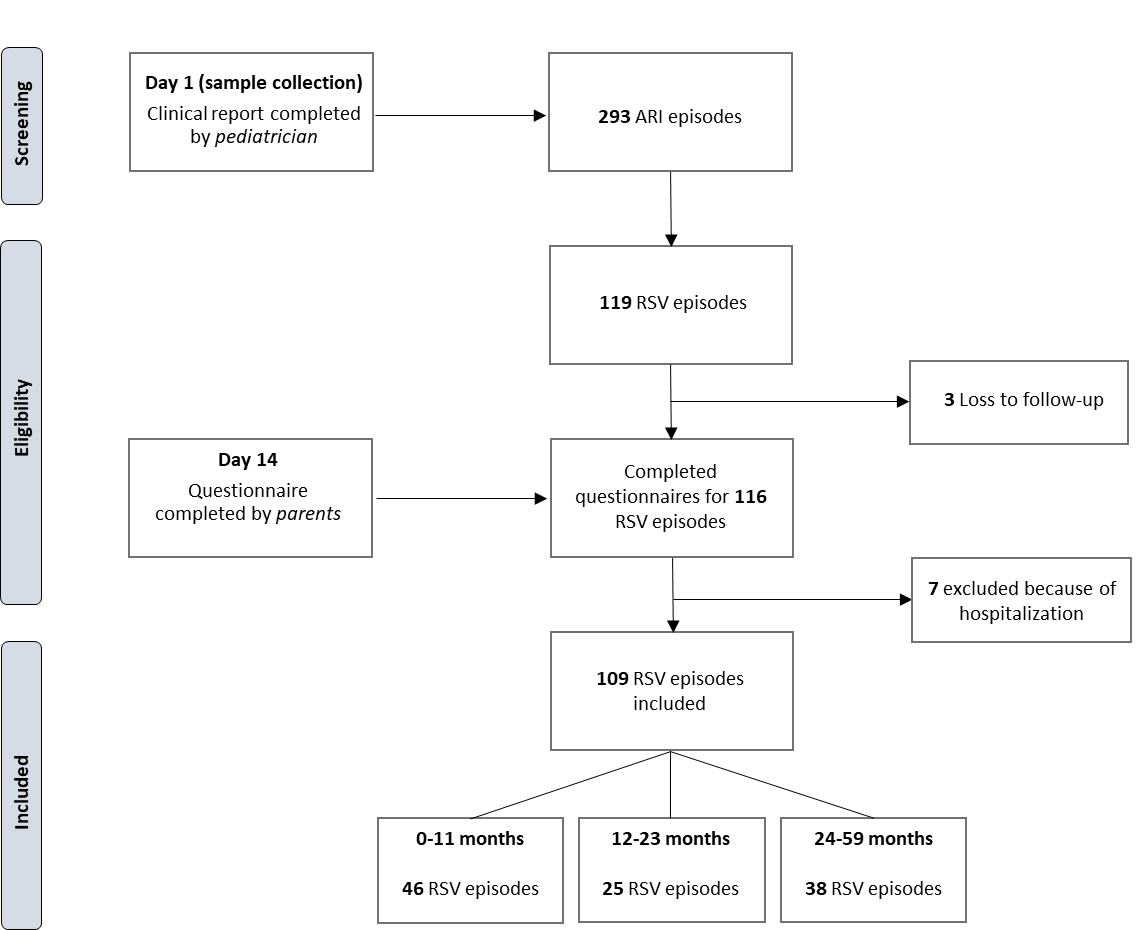


Abbreviations: ARI, acute respiratory infection; RSV, respiratory syncytial virus.
N.B.: in our study, each RSV-positive child represents a single RSV episode, meaning none of the included children experienced more than one RSV episode during our study period.

# **References**

1. Barbieri E, Porcu G, Petigara T, Senese F, Prandi GM, Scamarcia A, Cantarutti L, Cantarutti A, Giaquinto C: **The Economic Burden of Pneumococcal Disease in Children: A Population-Based Investigation in the Veneto Region of Italy**. *Children (Basel)* 2022, **9**(9).

2. Bozzola E, Ciarlitto C, Guolo S, Brusco C, Cerone G, Antilici L, Schettini L, Piscitelli AL, Chiara Vittucci A, Cutrera R *et al*: **Respiratory Syncytial Virus Bronchiolitis in Infancy: The Acute Hospitalization Cost**. *Front Pediatr* 2020, **8**:594898.

3. Italian Medicines Agency (AIFA). Available at: <https://www.aifa.gov.it/en/liste-di-trasparenza>. Accessed: March, 2023.

4. Garattini, L., Curto, A., & Padula, A. (2016). The puzzle of drug delivery in Italy: who wins? Expert Review of Pharmacoeconomics & Outcomes Research, 16(3), 331–332.

5. <https://www.farmacieravenna.com/>. Accessed: June, 2024.

6. Eurostat. Mean annual earnings. Available at: <https://ec.europa.eu/eurostat/databrowser/view/EARN_SES18_26__custom_2331746/default/table?lang=en>. Accessed: March, 2023.
